# Hope speech detection in YouTube comments

**DOI:** 10.1007/s13278-022-00901-z

**Published:** 2022-07-07

**Authors:** Bharathi Raja Chakravarthi

**Affiliations:** Insight SFI Research Centre for Data Analytics, Data Science Institute, National University of Ireland Galway, Galway, Ireland

**Keywords:** Hope speech, Equality, Diversity, Inclusion, Multilingual, Dravidian languages

## Abstract

Recent work on language technology has tried to recognize abusive language such as those containing hate speech and cyberbullying and enhance offensive language identification to moderate social media platforms. Most of these systems depend on machine learning models using a tagged dataset. Such models have been successful in detecting and eradicating negativity. However, an additional study has lately been conducted on the enhancement of free expression through social media. Instead of eliminating ostensibly unpleasant words, we created a multilingual dataset to recognize and encourage positivity in the comments, and we propose a novel custom deep network architecture, which uses a concatenation of embedding from T5-Sentence. We have experimented with multiple machine learning models, including SVM, logistic regression, K-nearest neighbour, decision tree, logistic neighbours, and we propose new CNN based model. Our proposed model outperformed all others with a macro F1-score of 0.75 for English, 0.62 for Tamil, and 0.67 for Malayalam.

## Introduction

Artificial intelligence is a rapidly evolving technology that significantly influences the global economy and society. Equality, Diversity, and Inclusion (EDI) have recently received a lot of attention, and an emphasis has been placed on protected classifications, including gender and race. Varied definitions of EDI may have different connotations depending on the context. EDI for an organization or workplace, for example, may have quite different priorities than EDI for a science community or a research subject, such as energy and AI. The emphasis may be on internal cultural changes in the workplace, with a small number of people serving as significant stakeholders. However, the larger academic community needs a much broader global perspective to define EDI and examine the entire society rather than individuals. The common core must be shared, and fair treatment must be assured, with equal opportunity for all and the abolition of all forms of discrimination. It began in 1960, but the definition of diversity has since expanded to include additional demographics such as lesbian, gay, bisexual, transgender, intersex, and queer/questioning (LGBTIQ+), women in science, engineering, technology, and management (STEM), and people with disabilities (Roberson et al. 2017).

Equality is defined as the fair and unbiased treatment of all individuals and groups. It is also expected that all groups would have an equal opportunity: the disadvantaged will have access to the same opportunities for advancement and accomplishment as their peers. Diversity is about being ’different’ and the manner in which it is expressed. It is often measured by the representation of people and groups from varied origins and viewpoints, but the essential idea is to acknowledge, accept, and celebrate such variety to stimulate creativity and innovation. Inclusion: An inclusive environment is one in which everyone can thrive and is valued. A diverse team has a number of points of view; inclusion ensures that those points are acknowledged and valued. In the research arena, it is equally crucial to have an inclusive approach to research and guarantee that research benefits all users, especially historically excluded communities (Roberson 2006; Shore et al. 2011; Xuan and Ocone 2022; Mehta et al. 2018). Nevertheless, the EDI for minority LGBTIQ+ or marginalized populations has not been considered with great urgency or importance compared to other topics or areas from the perspective of language technologies research (Cech and Waidzunas 2021). It is essential that language technologies are developed to consider the inclusion of all communities for social integration.

People have two images, one for the actual world they live in and another for the virtual world, such as the photos on social media platforms, where they are linked to close friends and converse with strangers in the virtual environment. Social networking services such as Instagram, Facebook, LinkedIn, and YouTube have become the default destination for individuals all around the world to spend their time. These social channels are utilized to not just share achievements but also request assistance in times of disaster. Many people’s lifestyles have been altered as a result of recent improvements in social media, and their everyday lives have been expanded with the virtual territory of the Internet and social networks. Social media platforms significantly impact users’ daily life. Users may post positive vibes, hope, or motivational information to provide positive proposals for peace or conquering problems such as COVID-19, conflict, or elections (Gowen et al. 2012; Yates et al. 2017; Wang and Jurgens 2018; Anderson et al. 2020). Several areas were affected worldwide, and the fear of losing a loved one caused the closure of even basic necessities such as schools, hospitals, and mental healthcare centers (Pérez-Escoda et al. 2020). Consequently, people were forced to look at online forums for their informational and emotional needs. In some areas, and for some people, online social networking was the only means of social connectivity and support during the COVID-19 pandemic (Elmer et al. 2020). As a result, individuals were driven to seek knowledge and emotional support from internet communities.

Online social networking provides a venue for network members to be in the know and to be known, both of which are more important with more social integration. Social inclusion is critical for the general well-being of all persons, particularly vulnerable individuals who are more vulnerable to social exclusion. A sense of belonging and community is an essential part of people’s mental health, influencing both their psychological and physical well-being (Rook and Charles 2017). People from marginalized groups, such as women in STEM fields, LGBTIQ+ people, people from racial minorities, and people with disabilities have been studied extensively, and it has been demonstrated that their online lives significantly impact their mental health (Chung 2013; Altszyler et al. 2018; Tortoreto et al. 2019). However, the contents from social media comments/posts may be negative/hateful/offensive/abusive, as there is no mediating authority.

Comments/posts on online social media have been analyzed to find and stem the spread of negativity using methods such as hate speech detection (Schmidt and Wiegand 2017), homophobia/transphobia detection (Chakravarthi et al. 2022c), offensive language identification (Zampieri et al. 2019a), and abusive language detection (Lee et al. 2018). However, according to Davidson et al. (2019), technologies developed for the detection of abusive language do not consider the potential biases of the dataset they are trained on. Because of persistent racial bias in the datasets, detection of abusive language is skewed. It may discriminate against one group more than another in some cases. This detrimentally influences people who are members of minority communities or are marginalized. As language is an essential component of communication, it should be accessible to everybody. A large internet community that uses language technology directly influences individuals worldwide, regardless of where they live. Instead of restricting an individual’s freedom of expression by eliminating unpleasant comments, we should direct our efforts to spreading optimism and encouraging others to do the same. On the other hand, hope speech detection should be carried out in conjunction with hate speech detection. Otherwise, hope speech recognition on its own may result in prejudice, and those who make harmful and damaging remarks on the Internet would continue to behave erratically.

Hence, we have shifted our study attention to the topic of hope speech. The promise, potential, support, comfort, recommendations, or inspiration offered to participants by their peers during moments of illness, stress, loneliness, and sadness are all typically connected with the term “hope” (Snyder et al. 2002). Psychologists, sociologists, and social workers in the Association of Hope have concluded that hope can also be a useful tool for saving people from committing suicide or harming themselves (Herrestad and Biong 2010). For example, the Hope Speech delivered by gay rights activist Harvey Milk on the steps of the San Francisco City Hall during a mass rally to celebrate California Gay Freedom Day on 25 June 1978^1^ inspired millions to demand rights for EDI (Milk 1997). Recently, Palakodety et al. (2020a) investigated how to utilize hope speech from social media texts to defuse tensions between two nuclear weapon-possessing states (India and Pakistan) and help marginalized Rohingya refugees (Palakodety et al. 2020b). Additionally, they experimented with identifying the presence of hope against its absence. Although no past research has been done on hope speeches for women in STEM, LGBTIQ+ folks, racial minorities, or people with disabilities in general, we believe that this area needs research.

Furthermore, despite the fact that people from a variety of linguistic origins are exposed to online social media language, English continues to be at the forefront of current developments in language technology research. Recently, various investigations have been carried out on languages with a lot of resources, such as Arabic, German, Hindi, and Italian, to name a few. The majority of such research relies on monolingual corpora and does not analyze code-switched textual data (Sciullo et al. 1986). We introduce a dataset for hope speech identification not only in English but also in under-resourced code-switched Tamil (ISO 639-3: tam), Malayalam (ISO 639-3: mal), and Kannada (ISO 639-3: kan) languages. We have experimented with multiple machine learning models, including SVM, logistic regression, K-nearest neighbour, decision tree, and logistic neighbours. Our proposed model outperformed all the others with a macro F1-score of 0.75 for English, 0.62 for Tamil, and 0.67 for Malayalam.

- We propose to encourage hope speech rather than take away an individual’s freedom of speech by detecting and removing a negative comment.
- We apply the schema to create a multilingual hope speech dataset for EDI. This is a new large-scale dataset of English, Tamil (code-mixed), and Malayalam (code-mixed) YouTube comments with high-quality annotation of the target.
- We have experimented with multiple machine learning models, including SVM, logistic regression, K-nearest neighbour, decision tree, logistic neighbours, and we propose new CNN based model. Our proposed model outperformed all the others with a macro F1-score of 0.75 for English, 0.62 for Tamil, and 0.67 for Malayalam.

## Related works

When it comes to crawling social media data, there are many works on YouTube mining (Marrese-Taylor et al. 2017; Muralidhar et al. 2018), mainly focused on exploiting user comments. Krishna et al. (2013) did an opinion mining and trend analysis on YouTube comments. The researchers analyzed sentiments to identify their trends, seasonality, and forecasts; user sentiments were found to be well-correlated with the influence of real-world events. Severyn et al. (2014) systematically studied opinion mining targeting YouTube comments. The authors developed a comment corpus containing 35K manually labeled data for modelling the opinion polarity of the comments based on tree kernel models. Chakravarthi et al. (2020a), Chakravarthi et al. (2020b), Sampath et al. (2022), Chakravarthi et al. (2022b), and B et al. (2022b) collected comments from YouTube and created a manually annotated corpus for the sentiment analysis, emotional analysis, offensive language identification, and multimodal sentiment analysis of the under-resourced Tamil and Malayalam languages.

Methods to mitigate gender bias in natural language processing (NLP) have been extensively studied for the English language (Sun et al. 2019). Some studies have investigated gender bias beyond the English language using machine translation to French (Vanmassenhove et al. 2018) and other languages (Prates et al. 2020). Tatman (2017) studied the gender and dialect bias in automatically generated captions from YouTube. Technologies for abusive language (Waseem et al. 2017; Clarke and Grieve 2017), hate speech (Schmidt and Wiegand 2017; Ousidhoum et al. 2019), and offensive language detection (Nogueira dos Santos et al. 2018; Zampieri et al. 2019b; Sigurbergsson and Derczynski 2020) are being developed and applied without considering the potential biases (Davidson et al. 2019; Wiegand et al. 2019; Xia et al. 2020). However, current gender de-biasing methods in NLP are insufficient to de-bias other issues related to EDI in end-to-end systems of many language technology applications, which causes unrest and escalates the issues with EDI, as well as exacerbating inequality on digital platforms (Robinson et al. 2020).

Counter-narratives (i.e., informed textual responses) are another strategy that has received the attention of researchers recently (Chung et al. 2019; Tekiroğlu et al. 2020). A counter-narrative approach was proposed to weigh the right to freedom of speech and avoid over-blocking. Mathew et al. (2019) created and released a dataset for counter-speech using comments from YouTube. However, the core idea of directly intervening with textual responses escalates hostility even though it is advantageous to the writer to understand why their comment/post has been deleted or blocked and then favorably change the discourse and attitudes of their comments. Thus, we diverted our research to finding positive information such as hope and encouraging such activities.

Deep neural network models based on transformers have been used to detect abusive remarks on Bangla social media Aurpa et al. (2021), Lucky et al. (2021). Pre-training language architectures such as BERT (Bidirectional Encoder Representations from Transformers) and ELECTRA (Efficiency Learning an Encoder that Classifies Token Replacements Accurately) are used in conjunction. The authors created a unique dataset, which consists of 44,001 comments from a large number of different Facebook posts in the Bangla language.

Our work differs from the previous works in that we define hope speech for EDI, and we introduce a dataset for English, Tamil, and Malayalam on EDI of it. To the best of our knowledge, this is the first work to create a dataset for EDI in the under-resourced Tamil and Malayalam languages. We also created a dataset for the Kannada language Hande et al. (2021) in continuation of this research.

## Hope speech

Hope is an upbeat state of mind based on a desire for positive outcomes in one’s life or the world at large, and it is both present- and future-oriented (Snyder et al. 2002). Inspirational talks about how people deal with and overcome adversity may also provide hope. Hope speech instills optimism and resilience, which beneficially impacts many parts of life, including (Youssef and Luthans 2007), college (Chang 1998), and other factors that put us at risk (Cover 2013). We define hope speech for our problem as “YouTube comments/posts that offer support, reassurance, suggestions, inspiration, and insight” (Chakravarthi 2020; Chakravarthi and Muralidaran 2021; Chakravarthi et al. 2022a).

The notion that one may uncover and become motivated to use routes to their desired goals is reflected in hope speech. Our approach seeks to shift the dominant mindset away from a focus on discrimination, loneliness, or the negative aspects of life toward a focus on creating confidence, support, and positive characteristics based on individual remarks. Thus, we provide instructions to annotators that if a comment/post meets the following conditions, it should be annotated as hope speech.

- The comment contains inspiration provided to participants by their peers and others and/or offers support, reassurance, suggestions, and insight.
- The comment promotes well-being and satisfaction (past), joy, sensual pleasures, and happiness (present).
- The comment triggers constructive cognition about the future – optimism, hope, and faith.
- The comment expresses love, courage, interpersonal skill, aesthetic sensibility, perseverance, forgiveness, tolerance, future-mindedness, praise for talents, and wisdom.
- The comment promotes equality, diversity, and inclusion.
- The comment brings out a survival story of gay, lesbian, or transgender individuals, women in science, or a COVID-19 survivor.
- The comment talks about fairness in the industry. (e.g., [I do not think banning all apps is right; we should ban only the apps that are unsafe.]).
- The comment explicitly talks about a hopeful future. (e.g., [We will survive these times.]).
- The comment explicitly talks about and says no to division in any form.

Non-hope speech includes comments that do not exude positivity, such as the following:

- The comment uses racially, ethnically, sexually, or nationally motivated slurs.
- The comment promotes hate towards a minority.
- The comment is very prejudiced and attacks people without thinking about the consequences.
- The comment does not inspire hope in the reader’s mind.

## Dataset construction

We concentrated on gathering information from comments on YouTube^2^, which is the most widely used platform in the world to comment and publicly express opinions about topics or videos. We didn’t include comments from LGBTIQ+ people’s personal coming out stories, as they contained personal information. For English, we gathered information on recent EDI themes such as women in STEM, LGBTIQ+ concerns, COVID-19, Black Lives Matter, United Kingdom (UK) vs China, the United States of America (USA), and Australia versus China. The information was gathered from recordings of individuals in English-speaking nations such as Australia, Canada, Ireland, the United Kingdom, the United States of America, and New Zealand.

**Fig. 1:**
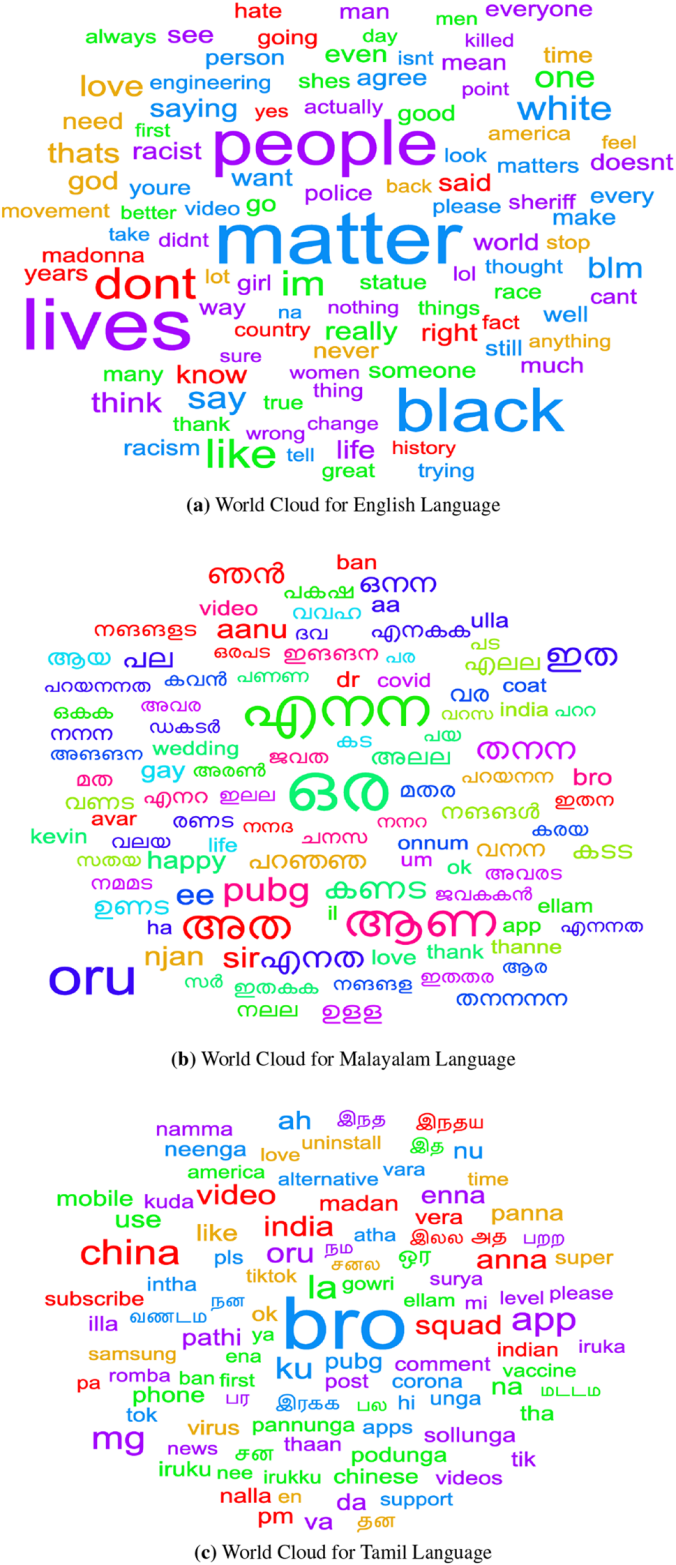
Language-Wise Word Cloud for the Dataset Without Pre-Processing

We gathered data from India for Tamil and Malayalam on recent themes such as LGBTIQ+ concerns, COVID-19, women in STEM, the Indo-China war, and Dravidian affairs. India is a multilingual and multiracial country. In terms of linguistics, India is split into three major language families: Dravidian, Indo-Aryan, and Tibeto-Burman. The ongoing Indo-China border conflict has sparked online bigotry toward persons with East Asian characteristics, despite the fact that they are Indians from the North-Eastern regions. Similarly, in Tamil Nadu, the National Education Policy, which calls for the adoption of Sanskrit or Hindi, has exacerbated concerns about the linguistic autonomy of Dravidian languages. We used the YouTube comment scraper^3^ to collect comments. From November 2019 to June 2020, we gathered data on the aforementioned subjects. We feel that our statistics will help reduce animosity and promote optimism. Our dataset was created as a multilingual resource to enable cross-lingual research and analysis. It includes hope speech in English, Tamil, and Malayalam, among other languages. The word cloud representation of the dataset is depicted in Fig. 1.

### Ethical concerns

Data from social media, especially data concerning minorities such as the LGBTIQ+ community or women, is extremely sensitive. By eliminating personal information from the dataset, such as names but not celebrity names, we have taken great care to reduce the danger of revealing individual identity in the data. However, to investigate EDI, we needed to track information on race, gender, sexual orientation, ethnicity, and philosophical views. Annotators only viewed anonymized postings and promised not to contact the author of the remark. Only researchers who agreed to follow ethical norms had access to the dataset for research purposes. After a lengthy debate with our local EDI committee members, we opted not to ask the annotator for racial information.^4^ Due to recent events, the EDI committee was firmly against the collection of racial information, based on the belief that it would split people on racial lines. Thus, we collected only the nationality of the annotators.

The information gathered via social media is particularly sensitive, especially pertaining to minorities, such as the LGBTIQ+ community or females. By removing personally identifiable information from the dataset, such as names but not celebrity names, we have made a great effort to minimize the possibility of individual identity being revealed in the data. It was necessary, however, in order to conduct an investigation into EDI to keep track of information about race and gender, sexual orientation, ethnicity, and philosophical viewpoints. Annotators were only allowed to examine anonymous remarks and were not allowed to contact the person who made the remark. Only researchers who agree to adhere to ethical standards will be granted access to the dataset for the purpose of conducting research. After a lengthy discussion with our local EDI committee members, we decided not to ask the annotator for racial information. We have defined the EDI community as people who are women in STEM fields, people who are members of the LGBTIQ+ community, people who are members of racial minorities, or people who are disabled for this study. EDI committee was vehemently opposed to the collection of race information because of previous occurrences, believing that it would cause individuals to be divided based on their racial origin. As a result, we only collected information on the nationality of annotators.

### Annotation setup

After the data collection phase, we cleaned the data using *Langdetect*^5^ to identify the language of the comments and removed comments that were not in the specified languages. However, owing to code-mixing at various levels, unintentional comments of other languages remained in the cleaned corpus of Tamil and Malayalam comments. Finally, based on our description from Section 3, we identified three groups, two of which are hope and non-hope, while the last (Other languages) was introduced to account for comments not in the required language. These classes were chosen because they provided a sufficient amount of generalization for describing the remarks in the EDI hope speech dataset.

### Annotators

We set up Google forms to collect annotations from annotators, which you can see below. Each form was restricted to 100 comments, and each page was limited to ten comments to maintain the level of annotation. Gender, educational background, and preferred medium of instruction of the annotator were all obtained to understand the annotator’s diversity and minimize prejudice in the annotation process. Those who participated in the annotation process were informed that the comments might contain profanity and hateful content. They had the option of discontinuing annotation if they found the comments too hurtful or burdensome. We educated annotators by introducing them to YouTube videos on electronic data interchange (EDI).^6^,^7^,^8^,^9^. Each form was annotated by at least three individuals. After the annotators marked the first form with 100 comments, the findings were manually validated as a warm-up phase. This strategy was utilized to help them gain a better knowledge of EDI and focus on the project. Following the initial stage of annotating their first form, a few annotators withdrew and their remarks were deleted. The annotators were requested to do another evaluation of the EDI videos and annotation guidelines. From Table 1, we can see the statistics of annotators. Annotators for the English language remarks came from Australia, Ireland, the United Kingdom, and the United States of America. We were able to obtain annotations in Tamil from persons from both India’s Tamil Nadu state and Sri Lanka. Graduate and post-graduate students constituted the majority of the annotators.

**Table 1:** Annotators

Language	English	Tamil	Malayalam
Gender			
Male	4	2	2
Female	5	3	5
Non-binary	2	1	0
Higher education			
Undergraduate	1	0	0
Graduate	4	4	5
Postgraduate	6	2	2
Nationality	Ireland, UK, USA, Australia	India, Sri Lanka	India
Total Total	11	6	7

### Inter-annotator agreement

We used the majority to aggregate the hope speech annotations from several annotators; the comments that did not get a majority in the first round were gathered and added to a second Google form to allow more annotators to contribute them. We calculated the inter-annotator agreement following the last round of annotation. We quantified the clarity of the annotation and reported on inter-annotator agreement using Krippendorff’s alpha. Krippendorff’s alpha applies to all these metrics. Our annotations achieved an agreement of 0.63, 0.76, and 0.85 for English, Tamil, and Malayalam, respectively, using the nominal measure. Previous research on sentiment analysis annotations and offensive language identification for Tamil and Malayalam in the code switched settings achieved 0.69 for Tamil, 0.87 for Tamil in sentiment analysis 0.74 for Tamil and 0.83 for Malayalam in offensive language. Our IAA values for hope speech are close to the previous research on sentiment analysis and offensive language identification in Dravidian languages.

### Corpus statistics

Our dataset contains 59,354 YouTube comments, with 28,451 comments in English, 20,198 in Tamil, and 10,705 in Malayalam. Our dataset also includes 59,354 comments in other languages. The distribution of our dataset is depicted in Table 2. When tokenizing words and phrases in the comments, we used the nltk tool to obtain corpus statistics for use in research. Tamil and Malayalam have a broad vocabulary as a result of the various types of code-switching that take place.

**Table 2:** Corpus statistic

Language pair	English	Tamil	Malayalam
Number of words	522,717	191,242	122,917
Vocabulary size	29,383	46,237	40,893
Number of comments/posts	28,424	17,715	9,817
Number of sentences	46,974	22935	13,643
Average number of words per sentences	18	9	11
Average number of sentences per post	1	1	1

Table 3 shows the distribution of the annotated dataset by the label in the reference tab: data distribution. Hence, the data is biased, with nearly all of the comments being classified as “not optimistic” (NOT). An automatic detection system that can manage imbalanced data is essential for being really successful in the age of user-generated content on internet platforms, which is increasingly popular. Using the fully annotated dataset, a train set, a development set, and a test set were produced.

**Table 3:** Class-wise Data Distribution

Class	English	Tamil	Malayalam
Hope	2,484	7,899	2,052
Not Hope	25,940	9,816	7,765
Total	28,451	17,715	9,817

A few samples from the dataset, together with their translations and hope speech class annotations, are shown below.

- **kashtam thaan. irundhaalum muyarchi seivom**-*It is indeed difficult. Let us try it out though.* Hope speech.
- **uff. China mon vannallo**-*Phew! Here comes the Chinese guy* Non-hope speech
- **paambu kari saappittu namma uyirai vaanguranunga**-*These guys (Chinese) eat snake meat and make our lives miserable* Non-hope speech

## Benchmark experiments

**Fig. 2:**
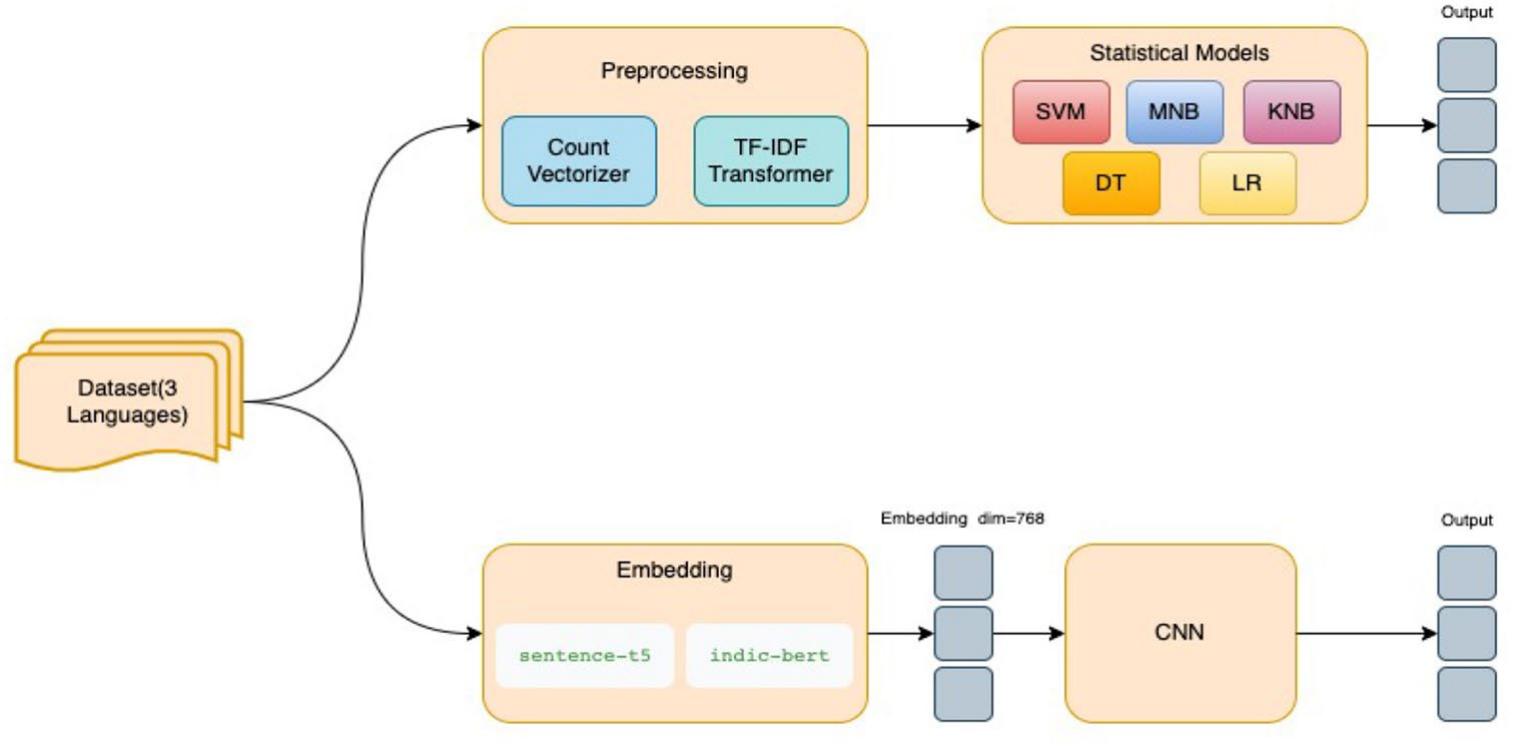
Experiment Flow Chart

We presented our dataset utilizing a broad range of common classifiers on the dataset’s imbalanced parameters, and the results were quite promising. The experiment was conducted on the token frequency-inverse document frequency (TF-IDF) relationship between tokens and documents. To generate baseline classifiers, we utilized the sklearn package (https://scikit-learn.org/stable/) from the sklearn project. Alpha = 0.7 was used for the multinomial Naive Bayes model. We employed a grid search for the k-nearest neighbors (KNN), support vector machine (SVM), decision tree, logistic regression, and decision tree and logistic regression (Fig. 2).

**Fig. 3:**
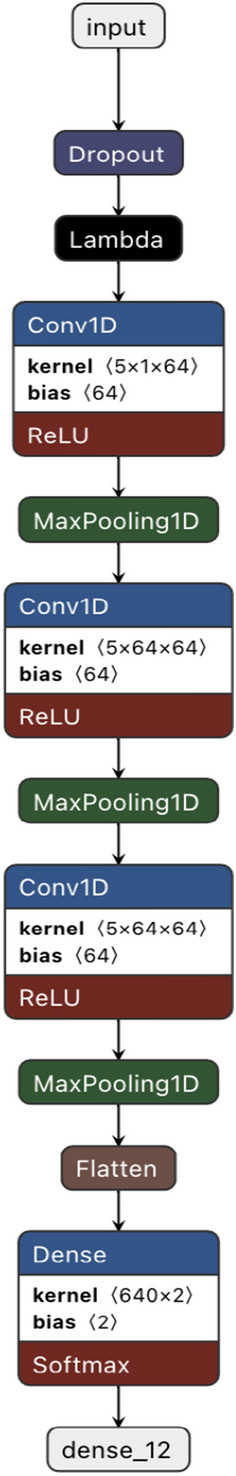
CNN Model: 1 Fully Connected Layer with 1536 Neurons with a ReLU Activation Function, Followed by a Dropout layer with dropout rate of 0.25, Followed by 3, 1-Dimensional Convolutional Layer with kernel size 5, 64 filters, and 1-D Max Pooling Layer of pool size 4, and the Last Layer-a Fully Connected Layer with 2 Neurons and Softmax Activation Function for Classification

We propose a novel custom deep network architecture, which will hereby be referred to as CNN, shown in Fig. 3, which uses a concatenation of embedding from T5-Sentence Ni et al. (2021). T5-Sentence is a sentence-embedding version of the T5  Raffel et al. (2019), which achieved state-of-the-art results on sentence representation learning benchmark SentGLUE, an extension using nine tasks from GLUE  Wang et al. (2018). Moreover, the CNN uses Indic-BERTKakwani et al. (2020), which is a pre-trained language model based on ALBERT Lan et al. (2019), trained on 11 major language of Indian origin, which is part of the on-going Indic NLP research, as the input for the deep net.

**Fig. 4:**
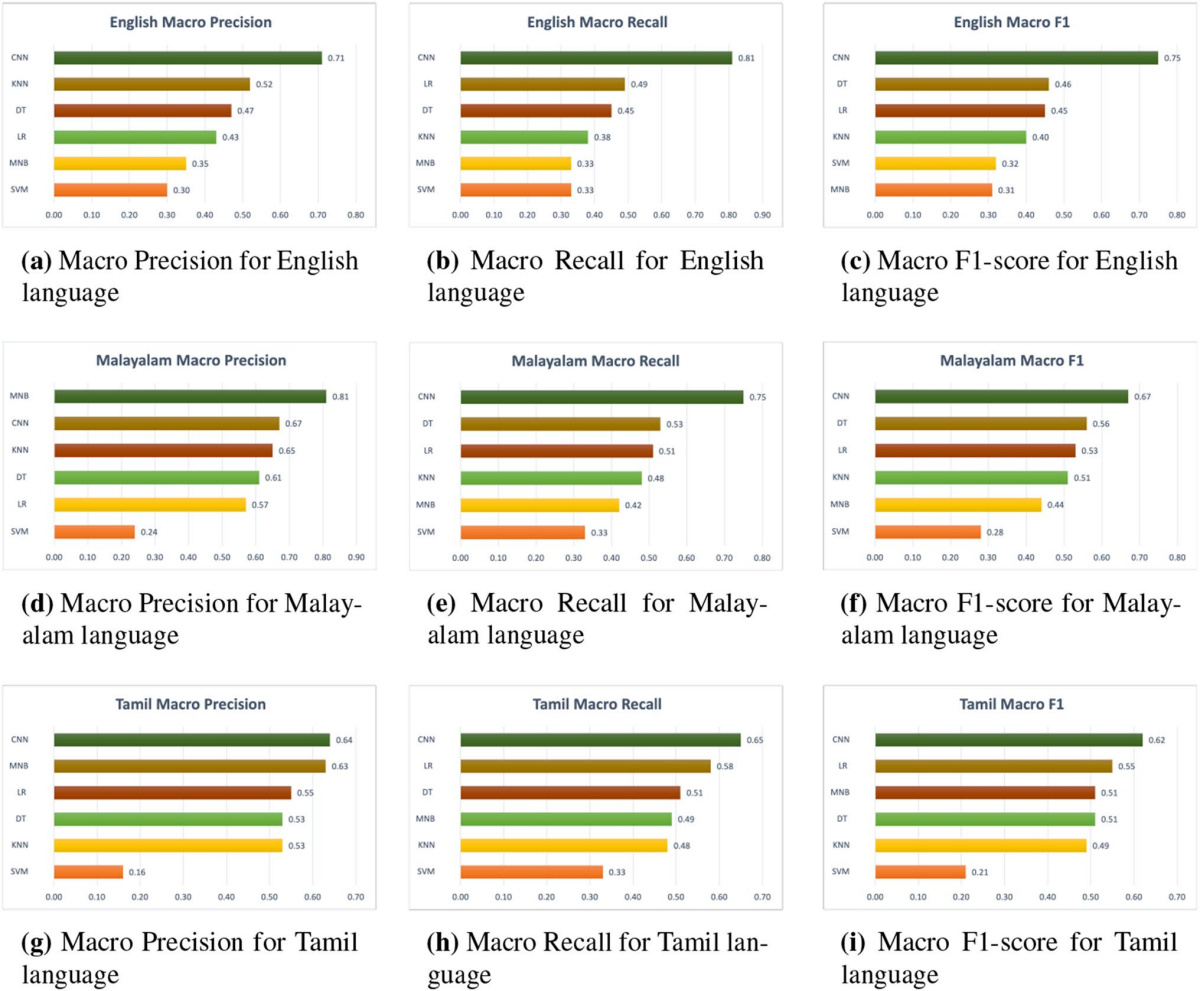
Macro Metrics of Different Models. SVM-Support Vector Machine, MNB-Multinomial Naive Bayes, KNN-K-Nearest Neighbour, DT-Decision tree, LR-Logistic Regression, CNN-Convolutional Neural Network(our proposed model)

**Fig. 5:**
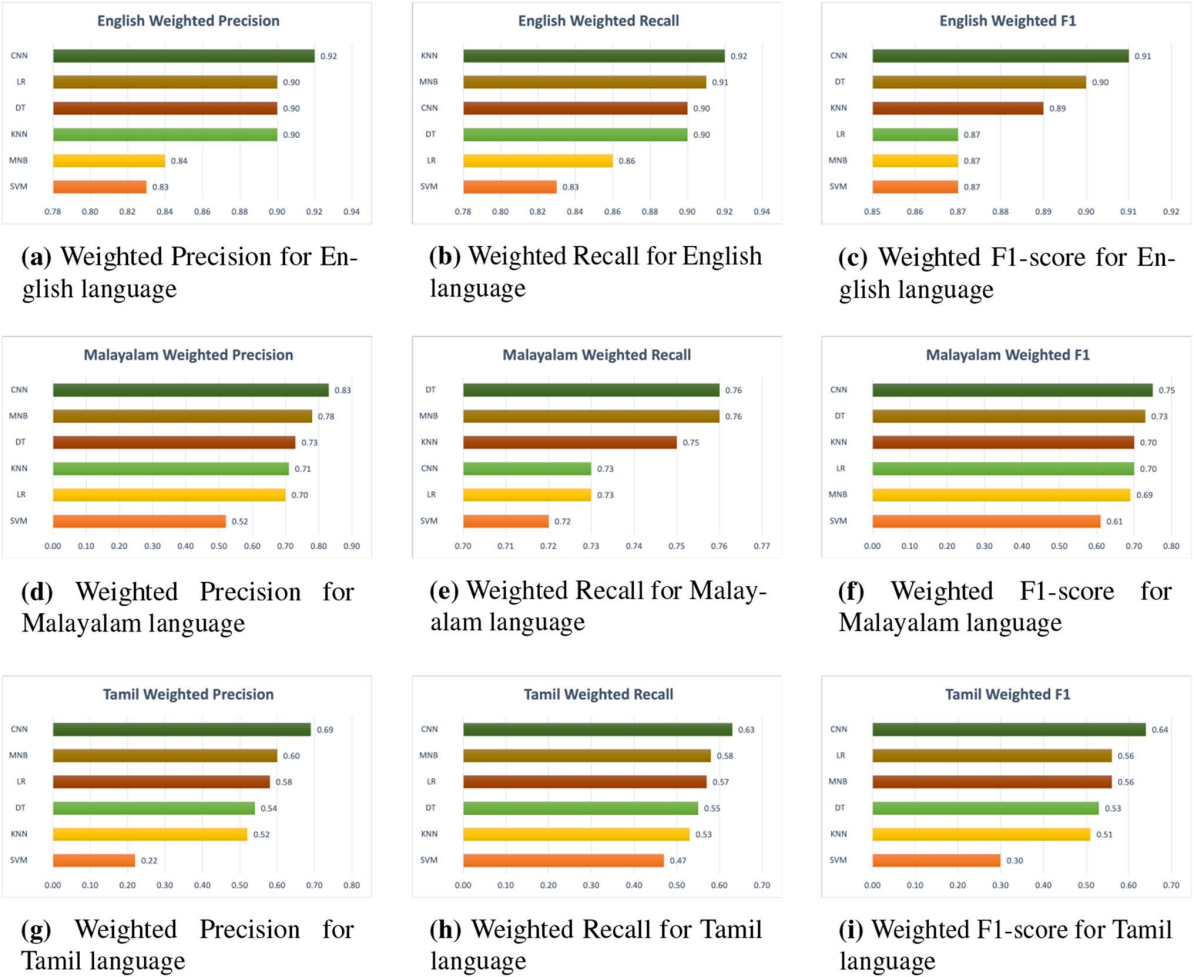
Weighted Metrics of Different Models. SVM-Support Vector Machine, MNB-Multinomial Naive Bayes, KNN-K-Nearest Neighbour, DT-Decision tree, LR-Logistic Regression, CNN-Convolutional Neural Network(our proposed model)

Using the training dataset, we trained our models; the development dataset was used to fine-tune the hyper-parameters, and the models were assessed by predicting labels for the held-out test set, as shown in Table 4. The performance of the categorization was measured using a macro-averaged F1-Score, which was derived by averaging accuracy and recall over a large number of trials. The motive behind this decision is the uneven class distribution, which causes well-known measures of performance such as accuracy and the micro-average F1-Score to be less than accurately representative of the actual performance. As the overall performance of all classes is important, we also presented the weighted-precision, weighted-recall, and weighted F1-Score of the individual courses in addition to the overall performance. The three tables in this section provide the precision, recall, and F1-Score findings of the HopeEDI test set, employing baseline classifiers in conjunction with support from test data: Tables 5, 6, and 7. The visual representation of the macro-precision, macro-recall and macro-F1 score of the proposed novel architecture model is available in Fig. 4. Similarly, the weighted-precision, weighted-recall and weighted-F1 are visualized as Fig. 5.

**Table 4:** Train-Development-Test Data Distribution

	English	Tamil	Malayalam
Training	22,735	13677	7,676
Development	2,843	2,018	1,070
Test	2,846	2,020	1,071
Total	28,424	17,715	9,817

**Table 5:** Precision, Recall, and F1-Score for English: Support is the number of actual occurrences of the class in the specified datase

Classifier	Hope Speech	Not-Hope Speech	Macro Avg	Weighted Avg
Support	250	2,593		
Precision				
SVM	0.23	0.91	0.30	0.83
MNB	0.24	0.91	0.35	0.84
KNN	0.63	0.92	0.52	0.90
DT	0.46	0.94	0.47	0.90
LR	0.33	0.96	0.43	0.90
CNN	0.45	0.97	**0.71**	**0.92**
Recall				
SVM	0.22	1.00	0.33	0.83
MNB	0.19	1.00	0.33	0.91
KNN	0.14	0.99	0.38	**0.92**
DT	0.39	0.96	0.45	0.90
LR	0.59	0.88	0.49	0.86
CNN	0.70	0.92	**0.81**	0.90
F1-Score				
SVM	0.21	0.95	0.32	0.87
MNB	0.20	0.95	0.31	0.87
KNN	0.23	0.96	0.40	0.89
DT	0.42	0.95	0.46	0.90
LR	0.43	0.92	0.45	0.87
CNN	0.55	0.94	**0.75**	**0.91**

Top values/high scores are highlighted in bold

**Table 6:** Precision, Recall, and F1-Score for Tamil. Support is the number of actual occurrences of the class in the specified datase

Classifier	Hope speech	Not-hope speech	Macro Avg	Weighted Avg
Support	815	946		
Precision				
SVM	0.00	0.47	0.16	0.22
MNB	0.58	0.57	0.63	0.60
KNN	0.48	0.55	0.53	0.52
DT	0.52	0.57	0.53	0.54
LR	0.59	0.59	0.55	0.58
CNN	0.48	0.80	**0.64**	**0.69**
Recall				
SVM	0.00	1.00	0.33	0.47
MNB	0.42	0.81	0.49	0.58
KNN	0.35	0.72	0.48	0.53
DT	0.40	0.71	0.51	0.55
LR	0.37	0.73	0.58	0.57
CNN	0.73	0.57	**0.65**	**0.63**
F1-Score				
SVM	0.00	0.64	0.21	0.30
MNB	0.49	0.67	0.51	0.56
KNN	0.41	0.62	0.49	0.51
DT	0.45	0.63	0.51	0.53
LR	0.46	0.65	0.55	0.56
CNN	0.58	0.67	**0.62**	**0.64**

Top values/high scores are highlighted in bold

As demonstrated, all of the models performed poorly because of an issue with class imbalance. Using the HopeEDI dataset, the SVM classifier had the worst performance, with a macro-average F1-Score of 0.32, 0.21, and 0.28 for English, Tamil, and Malayalam, respectively. The decision tree obtained a higher macro F1-Score for English and Malayalam than the logistic regression did; however, Tamil fared well in both tests. To eliminate non-intended language comments from our dataset, we applied language identification techniques. Other languages were annotated in some comments by annotators, although this was not the case in all of them. Another inconsistency was introduced into our dataset as a result of this. The majority of the macro scores were lower for English because of the “Other language” category. In the case of English, this could have been prevented by simply eliminating those comments from the dataset. However, this label was required for Tamil and Malayalam, as the comments in these languages were code-mixed and written in a script that was not native to the language (Latin script). The distribution of data for the Tamil language was roughly equal between the hope and non-hope classes.

The usefulness of our dataset was evaluated through the use of machine learning techniques, which we used in our trials. Because of its novel method of data collection and annotation, we believe that the HopeEDI dataset has the potential to revolutionize the field of language technology. We believe that it will open up new directions in the future for further research on positivity.

## Task Description

We also organized a shared task to invite more researchers toward hope speech detection and benchmark the data in LTEDI 2021 and LTEDI 2022 workshops.

Overall, we received a total of 31, 31, and 30 submissions for English, Malayalam, and Tamil tasks at LTEDI 2021. It is interesting to note that the top-performing teams in all the three languages predominantly used XLM-RoBERTa to complete the shared task. One of the top-ranking teams for English used context-aware string embeddings for word representations and recurrent neural networks and pooled document embeddings for text representation. Among the other submissions, although Bi-LSTM was popular, other machine learning and deep learning models were used. However, they did not achieve good results compared to the RoBERTa-based models (Tables 7, 8, 9, 10, 11, 12, 13).

**Table 7:** Precision, Recall, and F1-Score for Malayalam. Support is the number of actual occurrences of the class in the specified datase

Classifier	Hope speech	Not-Hope speech	Macro Avg	Weighted Avg
Support	194	776		
Precision				
SVM	0.00	0.72	0.24	0.52
MNB	0.78	0.76	**0.81**	0.78
KNN	0.39	0.77	0.65	0.71
DT	0.51	0.81	0.61	0.73
LR	0.46	0.79	0.57	0.70
CNN	0.40	0.93	0.67	**0.83**
Recall				
SVM	0.00	1.00	0.33	0.72
MNB	0.16	1.00	0.42	**0.76**
KNN	0.12	0.96	0.48	0.75
DT	0.27	0.92	0.53	**0.76**
LR	0.25	0.89	0.51	0.73
CNN	0.79	0.71	**0.75**	0.73
F1-Score				
SVM	0.00	0.84	0.28	0.61
MNB	0.26	0.86	0.44	0.69
KNN	0.19	0.86	0.51	0.70
DT	0.36	0.86	0.56	0.73
LR	0.33	0.84	0.53	0.70
CNN	0.53	0.81	**0.67**	**0.75**

Top values/high scores are highlighted in bold

**Table 8:** Rank list based on F1-score along with other evaluation metrics (Precision and Recall) for Tamil language

Team-Name	Precision	Recall	F1 Score	Rank
Spartans (Sharma and Arora 2021)	0.62	0.62	0.61	1
TeamUNCC (Mahajan et al. 2021)	0.61	0.61	0.61	1
NLP@CUET (Hossain et al. 2021)	0.61	0.61	0.6	2
Res-si sun	0.61	0.6	0.6	2
Team-hub (Huang and Bai 2021)	0.61	0.61	0.59	3
MUCS (Balouchzahi et al. 2021)	0.59	0.59	0.59	3
ZYJ (Zhao 2021)	0.59	0.59	0.59	3
Dhivya-hope-detection (Chinnappa 2021)	0.59	0.59	0.59	3
GCDH (Ziehe et al. 2021)	0.62	0.6	0.58	4
E8ijs	0.59	0.59	0.58	4
EDIOne-suman (Dowlagar and Mamidi 2021)	0.58	0.58	0.58	4
IIITK (Ghanghor et al. 2021)	0.58	0.58	0.58	4
HopeIsAllYouNeed	0.59	0.59	0.57	5
IRNLP-DAIICT-LR (Dave et al. 2021)	0.59	0.59	0.57	5
KBCNMUJAL	0.59	0.59	0.57	5
KU-NLP (M K and A P 2021)	0.62	0.6	0.56	6
Zeus (Zhou 2021)	0.59	0.59	0.56	6
CFILT-IITB-Submission	0.55	0.55	0.55	7
IIIT-DWD (Saumya and Mishra 2021)	0.54	0.54	0.54	8
Hopeful-nlp (Awatramani 2021)	0.57	0.56	0.53	9
MUM	0.53	0.53	0.53	9
Snehan-coursera	0.53	0.55	0.52	10
TeamX-Olawale Onabola	0.55	0.55	0.52	10
Hopeful-Men (Upadhyay et al. 2021)	0.52	0.55	0.49	11
SIMON (Que 2021)	0.63	0.56	0.49	11
Result	0.63	0.56	0.49	11
Amrita-CEN-NLP (S et al. 2021b)	0.48	0.49	0.47	12
MIGeng	0.42	0.42	0.42	13
Ssn-diBERTsity (S et al. 2021a)	0.43	0.44	0.38	14
IIITT-Karthik Puranik (Puranik et al. 2021)	0.38	0.39	0.37	15

**Table 9:** Rank list based on F1-score along with other evaluation metrics (Precision and Recall) for Malayalam language

Team-Name	Precision	Recall	F1 Score	Rank
NLP@CUET(Hossain et al. 2021)	0.86	0.85	0.85	1
MUCS (Balouchzahi et al. 2021)	0.85	0.85	0.85	1
GCDH (Ziehe et al. 2021)	0.84	0.85	0.85	1
ZYJ (Zhao 2021)	0.84	0.84	0.84	2
Team-hub (Huang and Bai 2021)	0.84	0.85	0.84	2
Res-si sun	0.84	0.85	0.84	2
KU-NLP (M K and A P 2021)	0.84	0.85	0.84	2
CFILT-IITB-Submission	0.84	0.85	0.84	2
TeamUNCC (Mahajan et al. 2021)	0.83	0.83	0.83	3
IIITK (Ghanghor et al. 2021)	0.83	0.84	0.83	3
HopeIsAllYouNeed	0.83	0.83	0.83	3
EDIOne-suman t (Dowlagar and Mamidi 2021)	0.83	0.83	0.83	3
E8ijs	0.83	0.84	0.83	3
Ssn-diBERTsity (S et al. 2021a)	0.82	0.81	0.81	4
Snehan-coursera	0.82	0.81	0.81	4
KBCNMUJAL	0.81	0.82	0.81	4
Hopeful-nlp (Awatramani 2021)	0.82	0.81	0.81	4
Dhivya-hope-detection (Chinnappa 2021)	0.81	0.82	0.81	4
IIIT-DWD (Saumya and Mishra 2021)	0.79	0.79	0.79	5
Zeus (Zhou 2021)	0.79	0.81	0.78	6
MUM	0.76	0.78	0.77	7
TeamX-Olawale Onabola	0.77	0.74	0.75	8
IRNLP-DAIICT-LR (Dave et al. 2021)	0.78	0.79	0.75	8
Hopeful-Men (Upadhyay et al. 2021)	0.76	0.79	0.75	8
Amrita-CEN-NLP (S et al. 2021b)	0.78	0.73	0.75	8
Amrita (S et al. 2021b)	0.76	0.72	0.73	9
Spartans (Sharma and Arora 2021)	0.62	0.62	0.61	10
MIGeng	0.58	0.61	0.59	11
IIITT-Karthik Puranik (Puranik et al. 2021)	0.57	0.57	0.57	12
SIMON (Que 2021)	0.63	0.56	0.49	13
Result	0.63	0.56	0.49	13

**Table 10:** Rank list based on F1-score along with other evaluation metrics (Precision and Recall) for English language

Team-Name	Precision	Recall	F1 Score	Rank
Zeus (Zhou 2021)	0.93	0.94	0.93	1
TeamUNCC (Mahajan et al. 2021)	0.93	0.94	0.93	1
Team-hub (Huang and Bai 2021)	0.93	0.93	0.93	1
Res-si sun	0.93	0.93	0.93	1
NLP@CUET(Hossain et al. 2021)	0.93	0.93	0.93	1
KU-NLP (M K and A P 2021)	0.92	0.93	0.93	1
Hopeful-men (Upadhyay et al. 2021)	0.93	0.93	0.93	1
GCDH	0.93	0.93	0.93	1
EDIOne-suman t (Dowlagar and Mamidi 2021)	0.93	0.94	0.93	1
Cs-english (Chen and Kong 2021)	0.93	0.94	0.93	1
Autobots (Gundapu and Mamidi 2021)	0.93	0.93	0.93	1
Hopeful-nlp (Awatramani 2021)	0.93	0.94	0.93	1
ZYJ (Zhao 2021)	0.92	0.93	0.92	2
Ssn-diBERTsity (S et al. 2021a)	0.91	0.93	0.92	2
MUCS (Balouchzahi et al. 2021)	0.92	0.93	0.92	2
IRNLP-DAIICT-LR (Dave et al. 2021)	0.92	0.93	0.92	2
IIITK (Ghanghor et al. 2021)	0.92	0.92	0.92	2
HopeIsAllYouNeed	0.92	0.93	0.92	2
Dhivya-hope-detection (Chinnappa 2021)	0.92	0.92	0.92	2
CFILT-IITB-Submission	0.92	0.93	0.92	2
Snehan-coursera	0.92	0.91	0.91	3
IIITT-Karthik Puranik (Puranik et al. 2021)	0.92	0.91	0.91	3
MUM	0.89	0.91	0.9	4
IIIT-DWD (Saumya and Mishra 2021)	0.9	0.91	0.9	4
E8ijs	0.91	0.92	0.9	4
Wrecking-crew	0.9	0.91	0.87	5
HopeFighters	0.83	0.91	0.87	5
Amrita-CEN-NLP (S et al. 2021b)	0.83	0.91	0.87	5
MlGeng	0.86	0.85	0.85	6
TeamX-Olawale Onabola	0.9	0.77	0.81	7
KBCNMUJAL	0.88	0.5	0.61	8

**Table 11:** Rank list based on Macro F1-score along with other evaluation metrics (Macro Precision, Recall and Weighted Precision, Recall and F1-score) for English language at LTEDI 2022

Team-name	M_P	M_R	M_F1	W_P	W_R	W_F1	Rank
IIITSurat	0.560	0.540	0.550	0.870	0.890	0.880	1
MUCIC (Gowda et al. 2022)	0.540	0.550	0.550	0.870	0.850	0.860	1
ARGUABLY	0.550	0.540	0.540	0.870	0.880	0.870	2
CIC (Balouchzahi et al. 2022)	0.540	0.530	0.530	0.860	0.870	0.870	3
LeaningTower (Muti et al. 2022)	0.530	0.530	0.530	0.860	0.870	0.870	3
CUNI-TIET	0.510	0.520	0.510	0.860	0.820	0.840	4
Ginius (Surana and Chinagundi 2022)	0.510	0.510	0.510	0.860	0.860	0.860	4
Ablimet	0.410	0.410	0.410	0.880	0.880	0.880	5
SSN_ARMM (Vijayakumar et al. 2022)	0.420	0.410	0.410	0.880	0.890	0.880	5
LPS (Zhu 2022)	0.420	0.410	0.410	0.880	0.890	0.880	5
SSNCSE_NLP (B et al. 2022a)	0.430	0.390	0.400	0.870	0.900	0.880	6
Error_english	0.440	0.390	0.400	0.880	0.900	0.890	6
SOA_NLP (Kumar et al. 2022)	0.460	0.370	0.380	0.880	0.910	0.880	7

**Table 12:** Rank list based on Macro F1-score along with other evaluation metrics (Macro Precision, Recall and Weighted Precision, Recall and F1-score) for Tamil language at LTEDI 2022

Team-name	M_P	M_R	M_F1	W_P	W_R	W_F1	Rank
Ablimet	0.300	0.340	0.320	0.390	0.460	0.420	1
LPS (Zhu 2022)	0.290	0.340	0.310	0.390	0.440	0.410	2
ARGUABLY	0.290	0.330	0.300	0.380	0.440	0.400	3
SSN_ARMM (Vijayakumar et al. 2022)	0.280	0.320	0.300	0.370	0.420	0.390	3
SSNCSE_NLP (B et al. 2022a)	0.280	0.330	0.300	0.370	0.440	0.400	3
CEN	0.280	0.330	0.300	0.370	0.440	0.390	3
SOA_NLP (Kumar et al. 2022)	0.280	0.320	0.290	0.360	0.430	0.380	4

**Table 13:** Rank list based on Macro F1-score along with other evaluation metrics (Macro Precision, Recall and Weighted Precision, Recall and F1-score) for Malayalam language at LTEDI 2022

Team-name	M_P	M_R	M_F1	W_P	W_R	W_F1	Rank
ARGUABLY	0.640	0.530	0.500	0.760	0.790	0.750	1
SSN_ARMM (Vijayakumar et al. 2022)	0.470	0.500	0.490	0.700	0.780	0.740	2
SOA_NLP (Kumar et al. 2022)	0.520	0.480	0.480	0.720	0.790	0.740	3
CEN	0.520	0.470	0.480	0.720	0.790	0.740	3
Ablimet	0.450	0.520	0.480	0.700	0.760	0.730	3
LPS (Zhu 2022)	0.450	0.490	0.470	0.690	0.760	0.720	4
SSNCSE_NLP (B et al. 2022a)	0.440	0.470	0.450	0.680	0.750	0.710	5
YUN111	0.310	0.340	0.320	0.560	0.600	0.580	6
MUCIC (Gowda et al. 2022)	0.310	0.320	0.310	0.560	0.580	0.570	7

The top scores were 0.61, 0.85, and 0.93 for Tamil, Malayalam, and English. The range of scores was between 0.37 to 0.61, 0.49 to 0.85, and 0.61 to 0.93 for Tamil, Malayalam, and English datasets, respectively, at LTEDI 2021. It can be seen that the F1 scores of all the submissions on the Tamil dataset were considerably lower than those of Malayalam and English. It is not surprising that the English scores were better, because many approaches used variations of pre-trained transformer-based models trained on English data. Due to code-mixing at various levels, the scores are naturally lower for Malayalam and Tamil datasets. Among these two, the systems submitted performed poorly on Tamil data. The identification of the exact reasons for the bad performance in Tamil requires further research. However, one possible explanation for this could be that the distribution of ‘Hope_speech’ and ‘Non_hope_speech’ classes is starkly different from those of English and Malayalam. In the remaining two classes, the number of non-hope speech comments was significantly higher than hope speech comments.

The total of submissions received for the classification of English, Tamil, and Malayalam datasets for LTEDI 2022 shared task were 13,7, and 9, respectively. An ensemble of several machine learning classifiers, such as logistic regression, multinomial Naive Bayes, random forest, and support vector machines received the most votes out of all of the other submissions. On the other hand, we found that the performances of the machine learning classifiers that were utilized for this shared task were somewhat less than the baseline performances of the ML models employed the previous year. Although LSTM, BiLSTM, and CNN were utilized, the performance of these models was not as satisfactory as that of the transformer-based models.

## Conclusion

As online content increases massively, it is necessary to encourage positivity, such as hope speech in online forums, to induce compassion and acceptable social behaviour. This paper presented the largest manually annotated dataset of hope speech detection in English, Tamil and Malayalam, consisting of 28,451, 20,198 and 10,705 comments, respectively. We propose a novel custom deep network architecture, which uses a concatenation of embedding from T5-Sentence. We have experimented with multiple machine learning models, including SVM, logistic regression, K-nearest neighbour, decision tree and logistic neighbours. Our proposed model outperformed all the models with a macro F1-score of 0.75 for English, 0.62 for Tamil, and 0.67 for Malayalam. We believe that this dataset will facilitate future research on encouraging positivity. We aim to promote research in hope speech and encourage positive content in online social media for EDI. In the future, we plan to extend the study by introducing a larger dataset with further fine-grained classification and content analysis.

## Footnotes

1 http://www.terpconnect.umd.edu/~jklumpp/ARD/MilkSpeech.pdf.

2 https://www.youtube.com/.

3 https://github.com/philbot9/youtube-comment-scraper.

4 We have considered women in the fields of STEM, people who belong to the LGBTIQ+ community, racial minorities, or people with disabilities as EDI community for this study.

5 https://pypi.org/project/langdetect/.

6 https://www.youtube.com/watch?v=C-uyB5I6WnQ &t=6s.

7 https://www.youtube.com/watch?v=UcuS5glhNto.

8 https://www.youtube.com/watch?v=hNeR4bBUj68.

9 https://www.youtube.com/watch?v=LqP6iU3g2eE.
